# Sweetpotato- and Cereal-Based Infant Foods: Protein Quality Assessment, and Effect on Body Composition Using Sprague Dawley Rats as a Model

**DOI:** 10.1371/journal.pone.0120121

**Published:** 2015-04-02

**Authors:** Francis Kweku Amagloh, Tracy Chiridza, Marie-Eve Lemercier, Anne Broomfield, Patrick C. H. Morel, Jane Coad

**Affiliations:** 1 Food Processing and Technology Unit, Department of Biotechnology, Faculty of Agriculture, University for Development Studies, Nyankpala, Ghana; 2 Institute of Food, Nutrition and Human Health, College of Health, Te Kura Hauora Tangata, Massey University, Palmerston North, New Zealand; 3 Institut Polytechnique LaSalle Beauvais, Beauvais, Cedex, France; 4 Institute of Veterinary, Animal and Biomedical Sciences, College of Sciences, Massey University, Palmerston North, New Zealand; TNO, NETHERLANDS

## Abstract

The Protein Digestibility Corrected Amino Acid Score (PDCAAS) of sweetpotato-based complementary foods (OFSP ComFa and CFSP ComFa) and cereal-based infant products (Weanimix and Cerelac) was assessed using 3 wk-old male Sprague Dawley rats weighing between 53–67 g as a model for human infants. Also, the effect of consumption of the infant formulations on lean mass, bone mass content and fat mass was evaluated by Dual-Energy X-ray Absorptiometry (DEXA) using 6 wk-old Sprague Dawley rats (initial weight, 206-229 g). The ComFa products and Weanimix are household-level formulations, and Cerelac is a commercial infant cereal. The true protein digestibility score for Cerelac was 96.27%, and about 1.8% (*P*<0.0001) higher than that for OFSP ComFa, CFSP ComFa and Weanimix. However, OFSP ComFa had the highest un-truncated PDCAAS by a difference of 4.1%, than CFSP ComFa, and about 20% difference compared with both the Weanimix and Cerelac. All the products investigated had PDCAAS greater than 70%, the minimum protein quality requirement for complementary foods. Among the rats assigned to the four formulations, their bone mass and fat mass composition were not significantly different (*P*=0.08 and *P*=0.85, respectively). However, the rats on CFSP ComFa had higher lean mass than those on Cerelac (321.67 *vs*. 297.19 g; *P*=0.03). The findings from the PDCAAS and the DEXA-measured body composition studies indicate that complementary foods could be formulated from readily available agricultural resources at the household-level to support growth as would a nutritionally adequate industrial-manufactured infant cereal. Nonetheless, it should be noted that the findings of our studies are based on an animal model.

## Introduction

The quality of complementary foods is among the major drivers if infants and young children nutriture is to be improved. In low- and middle-income countries particularly, because of the perennial problem of poverty, most families cannot afford nutritionally-adequate proprietary cereal-based complementary food. Thus, caregivers in resource-poor households may prepare porridge, for example, from cereals only or in combinations with legumes, for complementary feeding [1–4]. Until micronutrient-enriched foods are made available through an efficient food distribution channel and government subsidies, dietary diversification remains an important strategy for improving the nutritional status such as vitamin A of infants in low- and middle-income countries.

In this vein, household-level complementary food products were previously formulated from a cream-fleshed sweetpotato (CFSP) [5], and recently from an orange-fleshed sweetpotato (OFSP) [6] as a dietary source of vitamin A for infants in low- and middle-income countries. To our knowledge, there is only one study [7], in addition to our previous study [5], in which amino acid score was used to estimate the protein quality of sweetpotato-based complementary foods.

However, true digestibility estimated from rat bioassay when considered with the amino acid scores of the essential amino acid, referred to as Protein Digestibility Corrected Amino Acid Score (PDCAAS), gives a better predictor index for protein quality than the amino acid score only [8,9]. It has been argued by Schaafsma [10] that the PDCAAS is the appropriate method to assess the protein quality of food. It is recommended in the Codex Standard (CODEX STAN 074–1981, Rev. 1–2006) [11] that the minimum PDCAAS for complementary foods should be 70% compared to that of casein, which has a PDCAAS of 100% [12].

Therefore, evaluation of the protein quality of the formulated sweetpotato-based complementary foods, denoted as ComFa formulations/products, which could be alternatives to commonly used cereal-based products is warranted.

Also, investigation of body composition (lean mass, bone mass content and fat mass) could vitally contribute data for assessing the overall quality of the ComFa formulations as complementary food. The Dual-Energy X-ray Absorptiometry (DEXA) method has been shown to be an accurate, reliable and non-invasive method for determining the body composition of small animals [13].

The objectives of the present study were to (i) assess the PDCASS of two household-level sweetpotato-based complementary foods and two cereal-based infant products (including one commercial infant cereal); and (ii) evaluate the effect of the consumption of sweetpotato- and cereal-based infant foods on lean mass, bone mass content and fat mass by DEXA using Sprague Dawley weanling rats as a model for human infants.

## Materials and Methods

### Complementary foods

Household-level complementary foods formulated from orange- and cream-fleshed sweetpotato denoted as OFSP ComFa and CFSP ComFa respectively [6], and a maize-soyabean-groundnut blended food (Weanimix) [14,15] were evaluated for protein quality (Expt 1) and effect on growth (Expt 2) in 3 wk-old weanling and 6 wk-old Sprague Dawley rats, respectively. A commercial infant cereal (Nestlé Cerelac infant cereal wheat & *ikan bilis*), was included as a nutritionally-adequate product for infant feeding in both experiments.

The detailed method of preparing the ComFa formulations and Weanimix is published elsewhere [6]. Briefly, the composition of the ComFa products, on dry matter basis, was as follows: sweetpotato root (65%), full fat soyabean flour (8.0%), soyabean oil (7.0%) and anchovy powder (20%). To prepare 2 kg (dry matter basis), the ingredients were weighed (corrected for their initial moisture content), mixed with about 5 L of water and boiled for 2 h. The porridge obtained was freeze-dried before being used in these studies. Weanimix, which contained 75% maize, 15% soyabean and 10% groundnut [14,15] was processed as previously described [16]. For the two experiments, Weanimix slurry was prepared from the roasted flour and boiled for 2 h. The porridge obtained was freeze-dried; about 10 L of water were used for the preparation.

The Association of Official Analytical Chemists (AOAC) International protocols [17] for determination of moisture (AOAC 925.10), crude protein (total nitrogen × 6.25) (AOAC 960.25), crude fat (AOAC 922.06), ash (AOAC 969.32) and total dietary fibre (AOAC 991.43) were used to analyse the four formulations for the animal feeding experiments.

### Animal experimentation and ethics

Two experiments using 3 wk-old weanling and 6 wk-old Sprague Dawley rats as models for human infants were conducted to evaluate the protein quality (Expt 1) of the complementary food blends and their effect on body composition (Expt 2), respectively. The studies were approved (MUAEC 12/52) by the Animal Ethics Committee, Massey University, New Zealand.

### Expt 1: Assessment of protein quality using the PDCAAS

The macronutrient composition of the complementary food formulations is listed in Table 1 and was used to prepare iso-nitrogenous test diets (Table 2) as described in AOAC 991.29 [18]. Briefly, the freeze-dried samples of OFSP ComFa, CFSP ComFa and Weanimix were milled; afterwards, the pulverised samples as well as Cerelac (the commercial infant cereal) were each passed through 850-μm sieve and adjusted with other ingredients listed in Table 2.

**Table 1:** Proximate composition (g/kg, dry matter basis) of complementary food formulations*.

Complementary food^†^	Moisture	Crude Protein	Crude Fat	Ash	Crude fibre	Carbohydrates by difference
OFSP ComFa	72.66 (1.11)^a^	222.86 (2.79)^a,b^	116.08 (2.01)^a^	55.45 (0.49)^a^	122.76 (10.42)^a^	410.19 (12.05)^d^
CFSP ComFa	52.56 (1.69)^b^	208.90 (4.63)^b^	113.48 (0.84)^a^	55.08 (0.21)^a^	100.45 (1.77)^b^	469.53 (4.10)^c^
Weanimix	18.97 (0.35)^d^	230.20 (9.26)^a^	94.19 (0.54)^c^	35.36 (0.31)^b^	69.01 (6.44)^c^	552.26 (14.27)^b^
Cerelac	36.35 (0.37)^c^	154.27 (1.68)^c^	106.11 (0.72)^b^	30.50 (0.35)^c^	14.79 (4.97)^d^	657.98 (6.37)^a^

* Values are means and (standard deviations) of triplicate determinations; values (a-d) within a column that do not share the same letter are significantly different;

^†^ OFSP and CFSP ComFa, orange- and cream-fleshed sweetpotato-based blended infant food products; Weanimix, a maize-soyabean-groundnut complementary food; Cerelac, a proprietary infant dried cereal containing wheat, skimmed milk powder and fortified with micronutrients sourced from Nestlé, Malaysia.

**Table 2:** Test diet composition (g/kg, dry matter basis) for Expt 1.

Ingredient and proportion in test diet	OFSP ComFa	CFSP ComFa	Weanimix	Cerelac	Protein free diet
Test diet (10% protein)	448.6	478.7	434.4	648.1	0
Vitamin*(2%)	20.0	20.0	20.0	20.0	20.0
Mineral*(3.5%)	35.0	35.0	35.0	35.0	35.0
Cellulose powder^†^ (5% if test food contains less than 5% total dietary fibre)	0	1.9	20.0	40.4	50.0
Corn oil^‡^ (10%)	47.9	45.7	59.1	31.2	100
Wheaten corn starch^‡^ (to make up 100%)	448.5	418.7	431.5	225.3	795

*Vitamin and mineral composition: individual vitamin and mineral were sourced from Unitech Industries Limited, Auckland, New Zealand, and mixed based on the composition as in AIN-76 for rat as described in AOAC 991.29 [18];

^†^Sourced from Hawkins Watts, Auckland, New Zealand;

^‡^Sourced from Davis Trading, Palmerston North, New Zealand.

Thirty 3 wk-old male Sprague Dawley weanling rats weighing between 53–67 g as specified in the AOAC 991.29 protocol [18] were fed powdered standard laboratory chow during acclimatisation for 3 d. After acclimatisation, the rats were randomly allocated to five groups (*n* = 6 per group). They were housed in individual stainless steel cages, in a room maintained at an average temperature of 21°C and 66% relative humidity, alternating 12 h light and darkness cycles. The five groups were assigned either OFSP ComFa, CSFP ComFa, Weanimix, Cerelac or protein-free diet. The rats were fed 15 g daily of the assigned test diet provided in a stainless steel cup for a total of 9 d (for a 4-d preliminary period and a 5-day balance period) and Milli-Q water was provided *ad libitum* as specified in the FAO and AOAC protocols [9,18]. On each day of the 5-d balance period, food consumed was weighed. The spilled food and faeces were collected on 9 d and air-dried and separately weighed. The faeces were dried overnight in an oven, weighed, and ground for nitrogen determination by the Kjeldahl method (AOAC 960.25). At the end of the study, the rats were returned to the Small Animal Production Unit, Massey University, New Zealand.

The amino acid score, true protein digestibility and the PDCAAS were calculated for each test diet as described in the FAO and AOAC protocols [9,18]. The PDCAAS has been employed by other researchers to assess the protein quality of cereal-based foods for infants and young children [12,19].

### Expt 2: Body composition using DEXA study

Twenty-four 6 wk-old male Sprague Dawley rats weighing between 206–229 g were randomised into four groups (*n* = 6 per group), and fed milled freeze-dried OFSP ComFa, CSFP ComFa and Weanimix. Cerelac was fed as-received from the manufacturer. Unlike in Expt 1, the samples used in Expt 2 were not sieved in order to investigate body composition under similar food composition as would be served to babies. The rats were housed in individual stainless steel cages for 21 d, in a room maintained at an average temperature of 21°C and 66% relative humidity, with alternating 12 h light and darkness cycles. On day 1, each rat was given 15 g of the assigned test diets, followed by 18 g for days 2 to 6. From 7 d to the end of the study period, 25 g were provided daily. Milli-Q water was provided *ad libitum*. Food intake per group was measured daily throughout the study by weighing each food cup and subtracting this weight from the previously measured weight. Residue in the food cup was not discarded and the daily ration was added to it.

The body weight of each rat was measured weekly. On day 21, body weight as well as lean mass, bone mass content and fat mass under anaesthesia were measured using DEXA (Hologic, Discovery A, Quantitative Digital Radiography) with software (Hologic Apex Software, Version 13.3) for small animal analysis. For the *in vivo* DEXA scanning, the rats were anaesthetised with an i.p. injection at an overdose rate of 0.12 ml/100 g body weight using the injectable anaesthetic mixture: 2 mg/ml ACP (2 parts), 100 mg/ml Ketamine (5 parts), 10% Xylazine (1 part) and sterile water (2 parts). The rats were sacrificed by cardiac puncture using an 18 G needle and 10 ml syringe to withdraw blood, followed by a thoracotomy to ensure death had occurred.

### Statistical analysis

Data generated from both Expt 1 and Expt 2 were analysed by one-way ANOVA. Tukey's studentised range test was used to compare differences between means when the ANOVA was significant (*P* < 0.05). Minitab 16.2.2 (Minitab Inc., State College, PA, USA) was used for all the statistical analysis. All results are expressed as means and standard deviations.

## Results

### Expt 1: Assessment of protein quality using the PDCAAS

The macronutrient composition (Table 1) has been discussed elsewhere [6], and is only presented in this manuscript as it was used to formulate the test diets in Table 2. The FAO/WHO essential amino acid pattern for 6 mo- to 12 mo-old infants [20], concentrations of the essential amino acids in the complementary food products, and the calculation of the PDCAAS [9] are presented in Table 3. The contents of all the essential amino acids in the ComFa products were higher than the reference pattern for 6 mo- to 12 mo-old infant [20], but the lysine content in Weanimix and Cerelac were about 10% lower compared to the recommended content of 57 mg/g of protein. The sweetpotato-based infant food products, OFSP ComFa and CFSP ComFa, had limiting essential amino acid scores (lowest amino acid score ratio compared with the FAO/WHO amino acid pattern) above 1.0, while the cereal-based complementary foods (Weanimix and Cerelac) had scores of about 0.9. Leucine was the limiting essential amino acid in the sweetpotato-based products, but in the cereal-based infant foods, lysine was the limiting amino acid.

**Table 3:** The essential amino acid pattern, contents of essential amino acids in the infant food products, and the Protein Digestibility Corrected Amino Acid Score (PDCAAS) calculation*.

Essential amino acid	FAO/WH amino acid pattern^†^	OFSP ComFa	CFSP ComFa	Weanimix	Cerelac
	*mg/g protein*				
Histidine	20	33.24(3.16)^a^	33.53(4.10)^a^	33.40(5.89)^a^	29.51(1.62)^a^
Isoleucine	32	42.30(2.02)^a^	41.57(1.55)^a^	41.53(3.30)^a^	47.12(1.43)^a^
Leucine	66	72.19(3.34)^b^	69.75(2.92)^b^	90.30(8.56)^a^	87.77(2.46)^a^
Lysine	57	67.61(3.23)^a^	61.77(5.70)^a^	51.13(3.76)^b^	50.45(0.71)^b^
Methionine + Cysteine	28	40.00(1.38)^a^	41.63(4.75)^a^	43.72(2.52)^a^	46.09(1.15)^a^
Phenylalanine + Tyrosine	52	78.93(4.77)^b^	74.66(3.82)^b^	84.92(8.10)^a,b^	96.85(2.31)^a^
Threonine	31	41.97(2.48)^a^	40.55(1.16)^a^	38.55(3.65)^a^	38.45(1.44)^a^
Tryptophan	8.5	12.91(0.33)^b,c^	13.99(0.53)^b^	12.26(0.24)^c^	15.25(0.52)^a^
Valine	43	52.82(2.81)^a,b^	50.88(1.96)^b^	51.01(4.43)^b^	59.62(1.56)^a^
AAS^‡^		1.09(0.05)^a^	1.06(0.04)^a^	0.90(0.07)^b^	0.89(0.01)^b^
LEAA^§^		Leucine	Leucine	Lysine	Lysine
True digestibility^||^ (%)		94.63(0.65)^b^	93.98(0.94)^b^	95.05(0.10)^b^	96.27(0.79)^a^
PDCAAS, un-truncated		103.51^a^	99.33^b^	85.26^c^	85.21^c^
PDCAAS, truncated		100	99	85	85

* Values are means and (standard deviations) of triplicate determinations except PDCAAS, truncated values; values (a-c) within a row that do not share the same are significantly different;

^†^Reference essential amino acid pattern for 6 mo- up to 12 mo-old infant; FAO/WHO/UNU, 2007 [20];

^‡^Amino acid score = amino acid content of test protein/recommended essential amino acid pattern;

^§^Limiting essential amino acid; LEAA is the lowest amino acid score ratio compared to the FAO/WHO amino acid pattern;

^||^True digestibility = $\frac{I-F+Fk}{I}$ *x 100*; I, nitrogen intake from test diet; F, faecal nitrogen; F_k_, metabolic nitrogen loss

The true protein digestibility data show that the digestibility of protein in Cerelac was 1.8% (*P*<0.0001) higher than the household-level formulated products (OFSP ComFa, CFSP ComFa and Weanimix). Nonetheless, the un-truncated PDCAAS values ranked the OFSP ComFa higher, by a difference of 4.1%, than CFSP ComFa. Compared with both Weanimix and Cerelac, the OFSP ComFa was higher by a difference of about 20%. All the test diet formulation met that the minimum 70% PDCAAS required for complementary foods [12].

**Fig 1:**
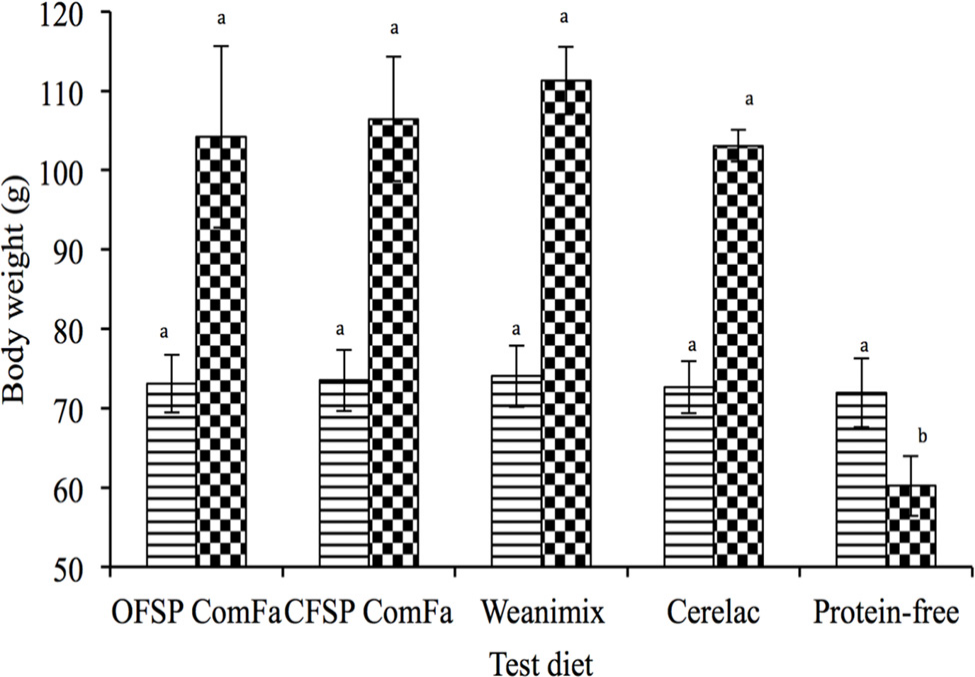
Body weight of the experimental groups (Expt 1): Striped bars represent day 1 and the checkered bars represent day 9. Bars represent group means and standard deviations for body weight. Bars for a particular day that do not share the same letter (a & b) are significantly different.

The mean body weights (Fig 1) on day one for Expt 1 were not significantly different (*P* = 0.91) between the groups. With the exception of the group on the protein-free test diet that lost about 12 g, the groups on the complementary food test diets gained on average, about 33 g (*P*>0.05) over the 9 d period.

### Expt 2: Body composition using DEXA analysis

**Fig 2:**
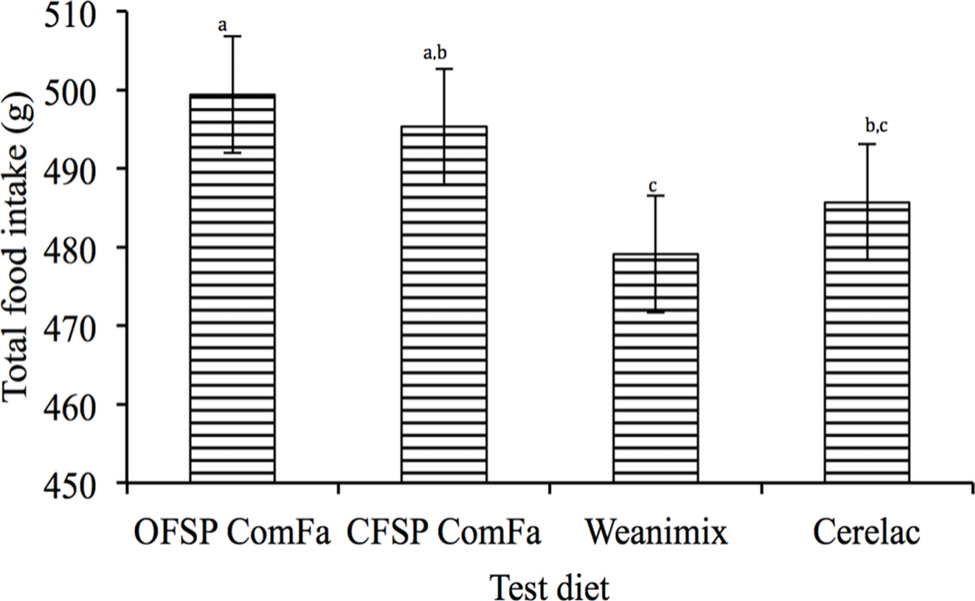
Total food intake for 21 d by experimental groups (Expt 2). Bars represent group means and standard deviations for total food intake. Bars that do not share the same letter (a-c) are significantly different.

The initial body weight of the groups were as follows: 216.82 g (OFSP ComFa), 218.66 g (CFSP ComFa), 216.05 g (Weanimix), and 215.42 g (Cerelac), and were not significantly different (*P* = 0.89). The initial body weight met the minimum recommended weight of 200 g suggested by Bertin et al [13] for obtaining reliable data of rats that are scanned on DEXA. All the groups gained an average weight of 156.46 g with no significant difference (*P* = 0.73) between them, although the total food intake differed (*P*<0.0001) between some of the groups (Fig 2). The OFSP ComFa group had the highest food intake and was not significantly different from the CFSP ComFa group (499.36 *vs*. 495.30; *P*>0.05). The total food intake by the Weanimix group was the lowest and not significantly different from the Cerelac group (479.11 *vs*. 485.69 g; *P*>0.05) (Fig 2).

**Fig 3:**
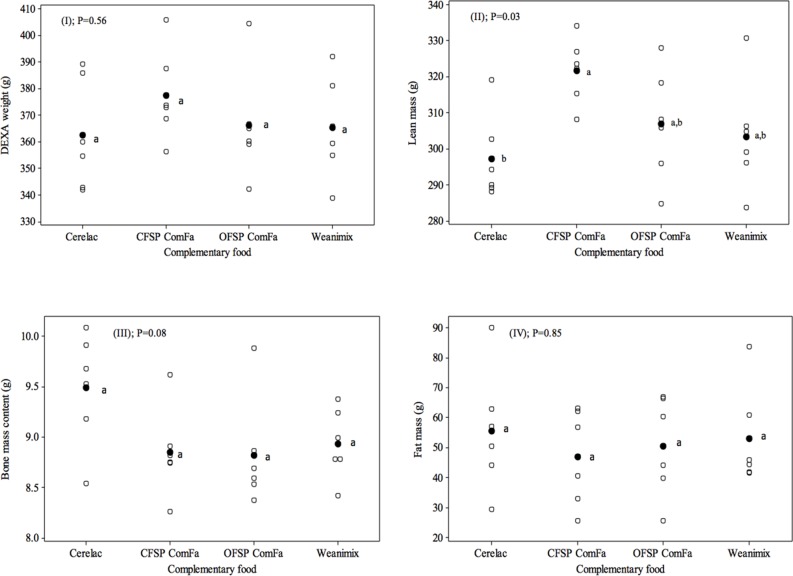
Body composition of experimental groups on the respective test diets at 21 d: (I) DEXA weight, (II) lean mass, (III) bone mass content and (IV) fat mass. ^●^Mean (n = 6) per test diet; Means that do not share the same letter (a & b) are significantly different.

Bone mass and fat mass composition were not significantly different (*P* = 0.08 and *P* = 0.85, respectively) between the groups on the four test diets (Fig 3, panels III and IV). However, the CFSP ComFa group had a significantly higher mean lean mass than only the Cerelac group (321.67 *vs*. 297.19 g; *P* = 0.03; Fig 3, panel II). The groups consuming the OFSP ComFa and Weanimix formulations had slightly higher mean lean mass than the Cerelac group (306.80 g and 303.35 g, respectively), but the difference was non-significant (*P*>0.05).

## Discussion

It has previously been reported that the vitamin A density of OFSP ComFa (about 226 μg retinol activity equivalents /100 kcal) [6], exceeded the vitamin A specification (60–180 μg retinol activity equivalents/100 kcal) in the Codex Standard for processed cereal-based foods for infants and young children [11]. In this study, the PDCAAS of the formulated sweetpotato-based complementary foods, and the effect of consumption of these formulations on body composition were investigated using Sprague Dawley rats as model.

In terms of protein quality, the OFSP ComFa and CFSP ComFa were the complementary foods that have limiting essential amino acid scores that meet the reference pattern for 6 mo- to 12 mo-old infants [20]. Nevertheless, all the complementary foods evaluated could be described as a source of quality protein to meet the demands of growth during infancy as the true protein digestibility values ranged between 93 and 100% [9]. Additionally the PDCAAS of all the products is greater than 70%, the minimum recommendation for complementary foods [11]. This PDCAAS rating indicates that the protein content of the complementary food formulations evaluated in this study would be more bioavailable to meet the growth demand of older infants. The high protein quality of the formulations in this study is due to the sources of protein, which are soyabean and fish in the household-level blends (OFSP ComFa, CFSP ComFa and Weanimix), and skimmed milk powder and fish in Cerelac. The limiting amino acid, lysine, found in the cereal-based products (Weanimix and Cerelac) was in agreement with findings by other researchers for other cereal-containing products assessed for their protein quality [12,21].

The PDCAAS of 85% for Weanimix (maize-soyabean-groundnut-fish blend) is higher than the 44 to 51% reported for maize-cowpea-soyabean or groundnut blend by Mensa-Wilmot and co-workers [21]. The difference is due to the inclusion of fish in the formulation we used. Therefore, if cereal-legume blends are to be recommended for complementary feeding, an animal source protein should be included. Needless to say, the OFSP ComFa described in this study, will be more appropriate than cereal-legume blends for household-level complementary feeding as it additionally meets the recommendation for daily consumption of vitamin A-rich food to complement breastfeeding [22]. Studies have shown that the consumption of OFSP improved vitamin A status of children [23,24].

The body composition analysis (Expt 2) of the present study shows no difference in the mean body weight, bone mass and fat mass between the groups at the end of the study when the DEXA analysis was done. Despite the slightly higher intake of food for the groups on the sweetpotato-based formulations than the cereal-based products, all would similarly support the growth of infants.

The lean mass measured tends to be higher for the groups on the household-level products (OFSP ComFa, CFSP ComFa and Weanimix) compared with Cerelac. A plausible reason for this pattern of growth could be the higher protein content in the household level products than the commercial product. The lack of a distinctive effect on body composition between the household-level products and commercial formulation could be explained in terms of their protein quality. All the formulation met the minimum PDCAAS of 70% recommendation in the Codex Standard [11].

A limitation of the body composition study (Expt 2) was that the commercial formulation donated was not sufficient to conduct the experiment for more than 3 wk. Hence, 3 wk-old weanling rats were not used based on the results of Expt 1 (weight gain after 9 days was 33 g on the average). A body weight of greater than 200 g was required to ensure that the rats would be appropriate to be scanned on DEXA [13].

Based on the findings of Expt 1 & 2, coupled with the benefits of feeding OFSP on vitamin A status [23–25], we conclude that the OFSP ComFa can positively contribute to the efforts aimed at reducing vitamin A deficiency prevalence in low-income countries. Ruel and Alderman [26] argued that it is only the use of OFSP (as an agricultural intervention programme) that had a positive effect on improving vitamin A status among women and children.

To our knowledge, data on assessment of protein quality of complementary food prepared from sweetpotato and the effect of its consumption on body composition have not been published elsewhere. Thus, our findings provide valuable preliminary data that will justify the relevance of conducting human infant studies for sweetpotato-based complementary food products. The strength of the present study is the inclusion of Cerelac, a fortified infant cereal manufactured by Nestlé, as a reference standard food.

To conclude, OFSP could be used to formulate complementary food to support growth to the same extent as nutritionally adequate industrial-manufactured infant cereal. However, it should be noted that the findings of our studies are based on an animal model; therefore validation of the findings using human infants is warranted.

## Supporting Information

S1 Data Raw data analyzed for figures and tables. (XLSX)

## References

[1] Mouquet C, Salvignol B, Van Hoan N, Monvois J, Treche S (2003) Ability of a "very low-cost extruder" to produce instant infant flours at a small scale in Vietnam. Food Chemistry 82: 249–255.

[2] Amuna P, Zotor F, Sumar S, Chinyanga Y (2000) The role of traditional cereal/legume/fruit-based multimixes in weaning in developing countries. Nutrition and Food Science: 116–122.

[3] Ejigui J, Desrosiers T (2011) Contribution to the improvement of a porridge made with fermented maize: Effect of selected foods and lemon on energy density, pH, viscosity and nutritional quality. International Journal of Food Sciences and Nutrition 62: 484–497. 10.3109/09637486.2010.547461 21366382

[4] Tizazu S, Urga K, Belay A, Abuye C, Retta N (2011) Effect of germination on mineral bioavailability of sorghum-based complementary foods. African Journal of Food, Agriculture, Nutrition & Development 11: 5083–5095.

[5] Amagloh FK, Hardacre A, Mutukumira AN, Weber JL, Brough L, Coad J (2012) A household-level sweet potato-based infant food to complement vitamin A supplementation initiatives. Maternal and Child Nutrition 8: 512–521. 10.1111/j.1740-8709.2011.00343.x 22145941 PMC6860577

[6] Amagloh FK, Coad J (2014) Orange-fleshed sweet potato-based infant food is a better source of dietary vitamin A than a maize-legume blend as complementary food. Food and Nutrition Bulletin 35: 51–59. 24791579 10.1177/156482651403500107

[7] Nnam NM (2000) Chemical evaluation of multimixes formulated from some local staples for use as complementary foods in Nigeria. Plant Foods for Human Nutrition (Formerly Qualitas Plantarum) 55: 255–263. 11030479 10.1023/a:1008176903750

[8] Sun M, Mu T, Zhang M, Arogundade LA (2012) Nutritional assessment and effects of heat processing on digestibility of Chinese sweet potato protein. Journal of Food Composition and Analysis 26: 104–110.

[9] FAO/WHO (1991) Protein quality evaluation; Report of the Joint FAO/WHO Expert Consultation; Food and Nutrition Paper. Rome, Italy: Food and Agriculture Organization of the United Nations. 51 51.

[10] Schaafsma G (2000) The protein digestibility-corrected amino acid score. Journal of Nutrition 130: 1865S–1867S. 10867064 10.1093/jn/130.7.1865S

[11] Codex Alimentarius Commission (2006) Codex standard for processed cereal-based foods for infants and young children. Rome, Italy: Codex Alimentarius Commission. CODEX STAN 074–1981, Rev. 1–2006 CODEX STAN 074–1981, Rev. 1–2006. 1–9 p.

[12] Serrem CA, de Kock HL, Oelofse A, Taylor JRN (2011) Rat bioassay of the protein nutritional quality of soy-fortified sorghum biscuits for supplementary feeding of school-age children. Journal of the Science of Food and Agriculture 91: 1814–1821. 10.1002/jsfa.4389 21448861

[13] Bertin E, Ruiz J-C, Mourot J, Peiniau P, Portha B (1998) Evaluation of dual-energy x-ray absorptiometry for body composition assessment in rats. Journal of Nutrition 128: 1550–1554. 9732318 10.1093/jn/128.9.1550

[14] Agble R (1997) Effective programmes in Africa for improving nutrition: Effective programmes for improving nutrition in Ghana. SCN News No. 15: 9–10. 12293189

[15] Lartey A, Manu A, Brown KH, Peerson JM, Dewey KG (1999) A randomized, community-based trial of the effects of improved, centrally processed complementary foods on growth and micronutrient status of Ghanaian infants from 6 to 12 mo of age. American Journal of Clinical Nutrition 70: 391–404. 10479202 10.1093/ajcn/70.3.391

[16] Amagloh FK, Hardacre A, Mutukumira AN, Weber JL, Brough L, Coad J (2012) Sweet potato-based complementary food for infants in low-income countries. Food and Nutrition Bulletin 33: 3–10. 22624294 10.1177/156482651203300101

[17] AOAC (2005) Official methods of analysis of AOAC International; Horwitz W, editor. Gaithersburg MD, Washington, DC, USA: AOAC International

[18] AOAC International (2006) AOAC official method 991.29: True protein digestibility of foods and food ingredients In: Horwitz W, editor. Official methods of analysis of AOAC International. Gaithersburg, MD, Washington, DC, USA: AOAC International

[19] Kannan S, Nielsen SS, Mason AC (2001) Protein digestibility-corrected amino acid scores for bean and bean−rice infant weaning food products. Journal of Agricultural and Food Chemistry 49: 5070–5074. 11600068 10.1021/jf010323u

[20] FAO/WHO/UNU Expert Consultation (2007) Protein and amino acid requirements in human nutrition; Report of a joint FAO/WHO/UNU expert consultation. Geneva, Switzerland: WHO Press. WHO Technical Report Series 935 WHO Technical Report Series 935. 18330140

[21] Mensa-Wilmot Y, Phillips RD, Hargrove JL (2001) Protein quality evaluation of cowpea-based extrusion cooked cereal/legume weaning mixtures. Nutrition Research 21: 849–857.

[22] Pan American Health Organization (PAHO), World Health Organization (WHO) (2003) Guiding principles for complementary feeding of the breastfed child In: Dewey KG, Lutter CK, Martines J, Daelmans B, editors. Division of Health Promotion and Protection/Food and Nutrition Program. Washington, D.C., USA: Pan American Health Organization.

[23] Low JW, Arimond M, Osman N, Cunguara B, Zano F, Tschirley D (2007) Food-based approach introducing orange-fleshed sweet potatoes increased vitamin A intake and serum retinol concentrations in young children in rural Mozambique. Journal of Nutrition 137: 1320–1327. 17449599 10.1093/jn/137.5.1320

[24] van Jaarsveld PJ, Faber M, Tanumihardjo SA, Nestel P, Lombard CJ, Benade AJS (2005) β–carotene-rich orange-fleshed sweet potato improves the vitamin A status of primary school children assessed with the modified-relative-dose-response test. American Journal of Clinical Nutrition 81: 1080–1087. 15883432 10.1093/ajcn/81.5.1080

[25] Hotz C, Loechl C, Lubowa A, Tumwine JK, Ndeezi G, Nandutu Masawi A, et al (2012) Introduction of β-carotene–rich orange sweet potato in rural Uganda results in increased Vitamin A intakes among children and women and improved vitamin A status among children. Journal of Nutrition. 10.3945/jn.111.151829 22875553

[26] Ruel MT, Alderman H (2013) Nutrition-sensitive interventions and programmes: how can they help to accelerate progress in improving maternal and child nutrition? The Lancet 10.1016/S0140-6736(13)60843-0: Epub ahead of print. 23746780

